# A Signal for Voice and Speech Abnormalities in Myalgic Encephalomyelitis/Chronic Fatigue Syndrome

**DOI:** 10.3390/jcm14144847

**Published:** 2025-07-08

**Authors:** Stephanie L. Grach, Jaime Seltzer, Diana M. Orbelo

**Affiliations:** 1 Division of General Internal Medicine, Mayo Clinic, Rochester, MN 55905, USA; 2 The Myalgic Encephalomyelitis Action Network, Santa Monica, CA 90401, USA; 3 Department of Otolaryngology, Head and Neck Surgery, Mayo Clinic, Rochester, MN 55905, USA

**Keywords:** myalgic encephalomyelitis/chronic fatigue syndrome, voice, post-exertional malaise

## Abstract

**Background/Objectives**: Patients with myalgic encephalomyelitis/chronic fatigue syndrome (ME/CFS) may report abnormalities in voice and speech; however, no formal research has been conducted in this area. **Methods**: An online mixed-methods survey was completed by 685 people with ME/CFS. A total of 302 respondents completed the qualitative component (44.09%). Questions assessed disease experience with ME/CFS and post-exertional malaise without prompting on specific symptoms. Within the qualitative results, a search of the terms “speech, voice,” “words,” and “speak” was conducted. **Results**: Excluding neurocognitive associations, colloquial phrases, and “speech therapy,” there were 38 mentions of the terms in the context of voice or speech changes across 28 unique qualitative survey responses (9.27%). **Conclusions**: A notable portion of respondents reported voice or speech changes when responding to open-ended qualitative questions about their disease experience. More research is needed regarding the implications of voice and speech anomalies in ME/CFS pathology and disease monitoring.

## 1. Introduction

Alterations in voice and speech appear in several neurologic and related disorders with links to autonomic and inflammatory processes [1,2]. For example, the majority of individuals with Parkinson’s disease will develop dysarthria (disordered speech), often with hypophonia (soft phonation) [3]. Multiple sclerosis manifests with frequent speech impairment, often with slow, imprecise speech with reduced loudness and pitch variability, while autonomic nervous system dysfunction and Shy Drager Syndrome/Multiple System Atrophy feature altered laryngeal control with reduced intensity ranges and frequency variations [4,5]. Individuals with autoimmune inflammatory myopathy exhibit a marked increase in muscular voice disorders (odds ratio = 4.503), and COVID-19 is associated with dysphonia both in the acute and chronic phases of the disease [2,6]. Together, these findings indicate that voice and speech changes can occur across conditions where neuroinflammation and dysautonomia may be involved.

Myalgic encephalomyelitis/chronic fatigue syndrome (ME/CFS) is a debilitating neurologic disorder characterized by multi-system symptom exacerbations following physical, cognitive, or sensory activities, known as post-exertional malaise (PEM). As a result of PEM, people with ME/CFS have substantially reduced functional capacity [7,8]. On average, individuals with mild ME/CFS are able to function at 50% of their pre-illness baseline, whereas individuals with severe and very severe disease are housebound and bedbound (less than 5% of baseline function), respectively [9]. In our clinical experience, voice and speech changes are reported by ME/CFS patients, particularly during PEM; however, this has not been formally studied.

## 2. Materials and Methods

A mixed-methods survey was distributed through an associated social media community page for individuals living with ME/CFS. The stated purpose of the survey was to improve the understanding of the lived experience of activity management in ME/CFS. The introduction page to the survey indicated that individuals could proceed with the survey if aged 18 and diagnosed with myalgic encephalomyelitis/chronic fatigue syndrome or long COVID with post-exertional malaise identified. Participants did not receive compensation for the study.

The study involved first a quantitative and then secondly a qualitative questionnaire. Study questions centered on the topic of activity management, with a focus on pacing. Questions in the qualitative section of the study were designed to capture general experiences with additional dedicated sections on topics such as living with severe ME/CFS, challenges and obstacles, attitudes of others, and work/school experiences as related to pacing.

The survey link remained open 1 month following the initial invitation. A total of 964 responses were recorded. With duplicate data, declined consents [2], and blank data removed, 685 responses remained. A break page was included at the end of the quantitative component, alerting survey-takers that they had completed the first portion; individuals could then choose to end the survey at that point or continue with the qualitative component. Of the 685 total responses, 302 responses completed the qualitative component of the survey (44.09% of responses).

Data analysis was performed on all answers submitted to qualitative questions. A search of the data for terms “speech,” “voice,” “words,” and “speak” was performed. “Speech therapy,” neurocognitive associations—for example, “difficulty finding words”—and use of colloquial language—for example, “speaking about…”—were excluded. Authors S.L.G. and D.M.O reviewed each response for inclusion and exclusion.

## 3. Results

Of the total respondents, 603 (264 qualitative section completers, or “full completers” (FCs)) reported assignment of female sex at birth, 75 (34 FCs) reported assignment of male sex at birth, and 7 (4 FCs) chose not to disclose or specified it as other. Furthermore, 542 of the total respondents (236 FCs) identified their gender as female, 64 (27 FCs) as male, and 76 (39 FCs) respondents indicated an identity other than male or female, including, but not limited to, genderqueer, non-binary, or gender-fluid. Age ranged from 18 to 80 years, with a mean and median age of 47 years. Also, 640 of the total respondents, including 279 FC respondents, were Caucasian (93.43%, 92.38%); 411 of total respondents, including 186 FCs, indicated that they were disabled/unable to work (60.00%, 61.59%).

Our search revealed 38 mentions of the included terms in the context of affected voice and speech function as part of the disease experience. These 38 mentions were contained within 28 unique qualitative survey responses, corresponding to 9.27% of responses mentioning voice or speech changes. A total of 105 mentions were excluded based on the criteria above, primarily due to cognitive associations and terms being part of colloquial phrases rather than being due to physical voice or speech dysfunction. Independent agreement was achieved for each response.

In total, 7 responses contained the term “voice,” 12 responses contained the term “speech,” 9 responses contained the term “words,” and 9 responses contained the term “speak.” One response included a mention of both “voice” and “speech,” totaling thirty-eight included responses. Examples of how the terms “voice,” “speech,” “words,” and “speak” were utilized in the included responses are as follows:

Voice: “My voice sounds raw”; “Hoarse throat with no voice”.

Speech: “Loss of speech”; “Sluggish speech slurs middle words”.

Words: “Unable to move to articulate words”; “Slurring my words”.

Speak: “Could only…speak 2–3 sentences”; “Inability to speak”.

In total, 15 responses contained the term “voice,” 4 responses contained the term “speech,” 62 responses contained the term “words,” and 24 responses contained the term “speak,” totaling 105 excluded responses. Examples of how the terms “voice,” “speech,” “words,” and “speak” were utilized in the excluded responses are as follows:

Voice: “People’s voices seem so much louder”; “I just hear a voice in my head that says to stop”.

Speech: “Hardest to process speech”; “Listening to speech and music”.

Words: “Difficulty finding words”; “Songs with words used to do me in”.

Speak: “Health professional speaking to family”; “I can better speak up and ask for accommodations”.

Specific questions associated with the included responses are listed with the identified search term(s) in Table 1.

## 4. Discussion

In our study, 9.27% of respondents spontaneously reported changes in voice or speech as part of their ME/CFS symptom experience, including during PEM exacerbations. Notably, these reports emerged without direct prompting, meaning they were not asked to discuss voice and speech directly. This suggests that voice and speech alterations may be a meaningful yet underrecognized component of ME/CFS. Given that voice and speech symptoms are rarely assessed outside of subspecialties such as otorhinolaryngology, speech language pathology, and behavioral neurology, their prevalence is likely underestimated, underreported, and potentially undertreated.

This spontaneous reporting of voice- and speech-related experiences may signal an emergent clinical phenomenon in ME/CFS. While voice and speech changes are acknowledged and, in some cases, well-described in other neurologic and inflammatory conditions, little formal research has explored these phenomena in infection-associated chronic illnesses like ME/CFS.

Preliminary evidence from related conditions supports the relevance of this domain. One pilot study of 27 patients with long COVID assessed the outcomes of a 10-week program consisting of biweekly 45 min online classes focused on mindfulness, breathing retraining, vocal exercises, and singing. Program feedback was reported to be very positive with associated improvements in breathing and general well-being, and notably, 14.3% of those meeting ME/CFS criteria prior to the intervention no longer met those criteria afterward [10].

Symptom improvement with vocal therapies in ME/CFS and related conditions suggests an important connection between the fields in regard to underlying pathology. There are also numerous aspects to voice and speech, including but not limited to prosody, phonation, and articulation, which may each offer individual insights into specific disease effects including dysautonomia and inflammation as previously mentioned [1,10]. Further studies are needed to gain an understanding of these potential associations.

Notably, artificial intelligence (AI)-based voice technology is rising as a potentially transformative tool in healthcare, offering non-invasive and accessible methods for disease detection and monitoring. Initial studies in several neurologic and cardiovascular diseases have already been conducted [11,12,13]. The application of this technology fits well with neurologic disease evaluation as voice and speech rely on coordinated motor control and fine sensorimotor integration, which can change with disease progression. Conditions associated with fatiguing effects and frailty also have the potential to be reflected in individuals’ acoustic speech signals [14,15].

With the growing interest in this area—including voice assistants, speech synthesis, and vocal biomarkers—there is a compelling opportunity to study and better understand conditions like ME/CFS, where vocal and speech changes may reflect underlying multisystem dysfunction. These technologies could help uncover non-invasive markers of disease progression, offering both diagnostic and therapeutic insights. However, given the prevalence of cognitive dysfunction in ME/CFS, it will be important to assess cognitive variables in voice AI processing as well [16].

In addition to excluding apparent use of the terms in cognitive or other irrelevant contexts, we attempted to organize term mentions into categories of voice and speech dysfunction, including but not limited to articulation, phonation, and fluency. However, this was ultimately deferred, as >50% of mentions lacked specificity to dependably categorize. For example, whereas one participant did go on to define “unable to speak” as meaning reduced volume and altered quality, another defined it as difficulty with articulation, indicating that the meaning of other responses stating “unable to speak” or similar could not be assumed by our team. This highlights the need for studies specifically geared at elucidating the full nature of voice and speech alterations in the setting of ME/CFS by use of directed questionnaires, voice analysis technology, or other means.

There were several other limitations of our study. One includes a lack of verification of specific diagnostic criteria for the study group, as while participants all self-identified as ME/CFS by proceeding with the study, we did not confirm how a diagnosis was given or by what criteria (such as, 1994 Fukuda Criteria, 2003 Canadian Consensus Criteria, 2011 International Consensus Criteria, versus 2015 Institute of Medicine Criteria). Demographically, the percentage of individuals identifying as an identity other than male or female was higher than the average in the general population of 1–4%, though the literature is lacking to determine whether this is reflective of or deviates from the ME/CFS population as a whole [17]. There were no healthy controls for this study as a qualitative narrative assessment of patient experience, though the experience of true post-exertional malaise and its effects are considered overall specific to this patient group. It is also important to acknowledge approximately half of the survey respondents did not proceed to the qualitative section, which could have been related to many factors (severity/lack of energy capacity to proceed, for example) and it is difficult to predict whether voice and speech abnormalities would have been more or less common in that sample.

## 5. Conclusions

**Figure 1:**
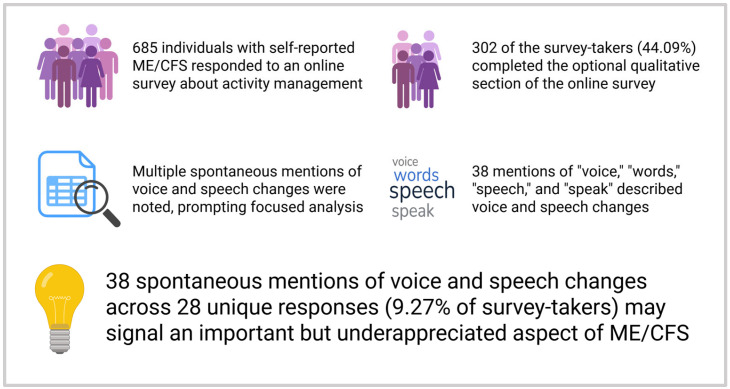
Summary schematic of the study. Created in BioRender. Grach, S. (2025) https://BioRender.com/wsnex4g (accessed on 6 July 2025).

In summary, our findings suggest that voice and speech abnormalities may represent an underappreciated but potentially meaningful manifestation of ME/CFS (Figure 1). Although our results from this study cannot quantify the specific prevalence of voice- and speech-associated symptoms in the disease, the use of open-ended responses to identify patient-reported symptoms without prompting was also the greatest strength of this study, offering insight into underreported experiences. The spontaneous emergence of these symptoms in qualitative narratives highlights a need for targeted investigation. Future research should systematically characterize the nature of these alterations, explore their mechanistic correlates, and assess their potential utility as diagnostic, prognostic, or therapeutic markers in ME/CFS and related conditions.

## Figures and Tables

**Table 1:** Questions to which survey participants responded with an answer indicating voice or speech dysfunction, delineated by search term as indicated by checkmarks.

Question	Voice	Speech	Words	Speak
What is pacing, as you define it?				√
If you had to pick one symptom to signify you were definitely going to crash/beginning to crash, what would it be?	√	√	√	√
Describe your response to overexertion before you started pacing.	√	√	√	√
Can you tell when you are approaching your limits? If so, what are the red flags you notice in your mind and body?			√	
Can you tell when you have overdone it and a crash is coming? If so, what are the red flags in your mind and body that tell you so?	√	√	√	√
What are the signs and signals that you are able to do less than usual on a particular day?	√	√		
Do the symptoms of your crash depend on the kind of trigger?		√		√
How do you decide how much you can do on a given day?		√		
What do you think was the toughest aspect of doing pacing/using pacing strategies?			√	
What are some coping mechanisms or strategies you use when you crash due to factors partially or mostly out of your control?				√
How do you communicate to others that your baseline has deteriorated?				√
Do you feel you pay a price for looking well in front of others?	√			
Is it possible to pace when you are housebound or bedbound? If so, what activities do you pace, and how?				√

## Data Availability

Data is currently unavailable as the initial phases of qualitative statistical analysis are still ongoing at this time. Individuals with specific questions about the data can reach out to the corresponding author at any time.

## Abbreviations

The following abbreviations are used in this manuscript:

ME/CFS	Myalgic Encephalomyelitis/Chronic Fatigue Syndrome
PEM	Post-Exertional Malaise
AI	Artificial Intelligence
